# Depression in an evolutionary context

**DOI:** 10.1186/1747-5341-3-8

**Published:** 2008-02-29

**Authors:** Lewis Wolpert

**Affiliations:** 1 Anatomy and Developmental Biology, University College, London, UK

## Abstract

Sadness and low levels of depression are adaptive since they lead the individual to try and make up a loss. By contrast, severe or clinical depression is not adaptive, but can be thought of as sadness having become malignant.

## 

Evolutionary thinking provides an account of the origin and function of most human characteristics.

That our genes control the development of our hands and brains so that we have the advantage to manipulate objects is quite clear. In evolution, changes in our body and behaviour due to changes in genes can result in the selection of those changes which are most favourable for survival and reproduction. Darwinian thinking about medicine can also help understand the origins of several illnesses [1]. The clearest example is the mutation that gives rise to sickle cell anaemia, which is more common in areas of malarial infection as it can protect against the infection because of the changed structure of the haemoglobin in red blood cells.

Depression is an emotional state, and if we are to understand depression in relation to evolution, we must first consider emotions. A common feature among different emotional states is that they are a response to signals, both external and internal, that are linked to positive reward or harm. They have evolved to make the individual survive better. Fear is an emotion that helps an individual escape from a dangerous situation, while pleasure encourages an individual to try and repeat the actions, like sex, that gave rise to it. Similar arguments can be made for other emotions like anger, disgust and sadness.

Sadness is a key emotion in relation to depression as it is the emotion most closely linked to depression. Sadness is a universal emotion, and has a common facial expression associated with it that is recognised in many different cultures [2]. Sadness is usually caused by loss of some sort, from a person to money. Sadness is closely linked to a loss of attachment to a child or to a partner, relative or close friend [3]. Attachment is adaptive from an evolutionary viewpoint, particularly in relation to the bond between mother and child, and loss of this attachment, even briefly, can cause sadness in young children and causes them to search for the parent. Attachment is also important for couples and its loss promotes sadness and the search for the partner. Sadness can also result from other losses, ranging from money to lack of success at work, and its biological and evolutionary function is to motivate the individual to recover what has been lost. Sadness drives us to restore attachment and is from an evolutionary point of view an important adaptive emotion. The sadness caused by bereavement is the cost of having been attached, and it may also act as a social signal that is a plea for sympathy

There is no clinical test for depression. Diagnosis is usually based on DSM-IV, the standard manual for diagnosing mental illnesses. Depression diagnosis is based on the presence of several of the following over two weeks: depressed mood most of the day; gain or loss of weight; too little or too much sleep; fatigue; thoughts of death or suicide; inability to concentrate; and guilt or feelings of worthlessness. But diagnosis based on these criteria may not distinguish low mood or sadness from a genuine clinical condition. This has been emphasised by Horwitz and Wakefield [4] who argue that depression as a clinical condition is much over diagnosed. Personal and cultural differences lead to different symptoms, including a variety of physical symptoms.

Many triggers for sadness and depression result from loss of some sort and should lead to making up the loss or accepting it. However it is important to realise the complexity of the emotion, as purely biological factors can trigger depression. Excess cortisol can be a cause [5], as can components of the immune system. If patients are given alpha interferon for hepatitis, they are give an antidepressant at the same time to stop them getting depressed [6]. Of even greater significance is the genetic component, which can be quite high as heritability is around 50% [7].

'Depression' has often been described as a most unsatisfactory term for the condition. A study found that the two terms generally associated with depression are sadness and grief. Depression does vary along a lumpy continuum from low mood or sadness to clinical depression. Just feeling low or feeling sad is not that different from what could be called a low level of depression. By contrast severe, or clinical, depression is very hard to describe. Everything is perceived as being negative, one believes that one will not recover, there are several negative physical symptoms, and suicidal thoughts are common.

About 10% of the population may suffer from a severe depression at some stage in their lives, yet there are no good descriptions of severe or clinical depression in English novels, even though several leading writers have themselves suffered from depression. However some writers, notably William Styron, in Darkness Visible [8], have given impressive accounts of their own depression. Clinical depression is a strange state and I have claimed that if you can describe your severe depression, you haven't truly experienced one [9].

When one suffers from a severe or clinical depression it is not possible to function in a normal way, and patients usually stop working. Severe depression is disabling not only in terms of day to day functioning but also because of the effects it can have on one's health – heart problems, for example are made much worse, and there are other symptoms such as pain in various organs [10]. So why has evolution not selected it out, particularly as suicide is all too common and it has a significant genetic component? Does depression arise from a defect in the body, or is it a defensive response, like pain? Is it adaptive from an evolutionary viewpoint?

It is hard for many to believe that so common a state does not have some advantage for the individual. There is no doubt that sadness is an adaptive emotion, but it is severe sadness, severe depression, that raises problems. Several hypotheses have been proposed to show that depression can have an advantage for the individual. One of the first was the social competition hypothesis [11,12]. This sees depression as an adaptation whose function is to inhibit aggression by rivals and superiors when one's status is low. It is a means of yielding when there is social competition and thus reduces the efforts by the aggressor. It is hard to see how this could actually function in any current human society, and why depression should be so physically and psychologically debilitating. In terms of these ideas, just giving in would be sufficient. It also is completely at variance with women having twice the incidence of depression as men, depression in children, and the increased chance of a depression in adulthood if a child is abused or neglected – all these argue against depression being used to yield to social competition, and being adaptive in this way.

Another hypothesis is that the function of depression, and of low mood, is to make people accept unobtainable goals and so change those goals [13,14]. It has also been proposed that it can lead to a conservation of energy in difficult circumstances [15]. This may make sense for low mood, but not for severe depression.

Another view, particularly in relation to postpartum depression, is that it is essentially a plea for help to the woman's partner in looking after the newborn child [16]. The social navigation hypothesis is that low mood and depression focuses resources and motivates partners to help [17]. Yet another approach is that varied situations can cause non-severe depression and that the symptoms serve related but distinguishable functions [18]. For example, sadness would result from loss, whereas crying may be a social signal, and fatigue reflects physical or mental weariness. But all these really deal with sadness at a low to moderately high level, and offer no evolutionary explanation for clinical and disabling depression [19].

A different approach to depression is to view it as sadness having become excessive and out of control, in other words, malignant [9]. Cancer is an example of a normal healthy process, cell multiplication, going wrong and becoming malignant. Cancer has its origin in a single cell with a small defect that then goes through a series of stages that lead to malignancy. The same may be true for depression in the sense that there is a normal process that has become disordered [4]. It may be that because sadness is a complex emotion it may increase to a malignant state due to loss of normal controls. The complexity of the processes involved may have prevented the evolution of adequate preventive mechanisms. Severe depression may result from the interaction of natural biological sadness and negative cognition – malignant sadness. There may be a positive feedback loop between the biological basis of sadness and the psychological basis that leads to severe depression. There is probably a contribution by other factors, such as genetic disposition and cytokines produced by the immune system.

Seasonal affective depression, SAD, occurs in winter in individuals who are normal during the rest of the year, and may be partly caused by lack of light, and it is more common northern countries. It could be an adaptation to reducing activity in the cold dark, months. There is increased eating and sleeping. Women are much more affected.

Since severe depression is not adaptive it must be treated as a severe illness.

## Competing interests

The author(s) declare that they have no competing interests.

## References

[B1] Nesse RM, Williams GC (1995). Evolution and Healing.

[B2] Izard CE (1991). The psychology of emotions.

[B3] Bowlby J (1981). Attachment and Loss, Loss: Sadness and Depression.

[B4] Horwitz AV, Wakefield JC (2007). The Loss of Sadness: How Psychiatry Transformed Normal Sorrow into Depressive Disorder.

[B5] Carroll BJ, Cassidy F, Naftolowitz D, Tatham NE, Wilson WH, Iranmanesh A, Liu PY, Veldhuis JD (2007). Pathophysiology of hypercortisolism in depression. Acta Psychiatr Scand.

[B6] Schiepers OJ, Wichers MC, Maes M (2005). Cytokines and major depression. Prog Neuropsychopharmocol Biol Psychiat.

[B7] McGuffin P, Katz R, Watkins S, Rutherford J (1996). A hospital based twin register of heritability of DSM-IV unipolar depression. Arch Gen Psychiatry.

[B8] Styron W (1990). Darkness Visible: A Memoir of Madness.

[B9] Wolpert L (2006). Malignant Sadness. The Anatomy of Depression.

[B10] Katona C, Peveler R, Dowrick C, Wessely S, Feinmann C, Gask L, Lloyd H, de C Williams A, Wager E (2005). Pain symptoms in depression: Definition and clinical significance. Clin Med.

[B11] Price J, Sloman L, Gardner R, Gilbert P, Rohde P (1994). The social competition hypothesis of depression. Brit J Psychiatry.

[B12] Price JS (1998). The adaptive function of mood change. Br J Med Psychol.

[B13] Hamburg DA, Hamburg BA, Barchas JD, Levi L (1975). Anger and depression in perspective of behavioral biology. Emotions: their parameters and measurement.

[B14] Klinger E (1975). Consequences of commitment to and disengagement from incentives. Psychol Rev.

[B15] Kaufman IC, Rosenblum LA (1969). The reaction to separation in infant monkeys: Anaclitic depression and conservation withdrawal. Psychosom Med.

[B16] Hagen EH (1999). The function of postpartum depression. Evol Hum Behav.

[B17] Watson PJ, Andrews PW (2002). Toward a revised evolutionary adaptationist analysis of depression: The social navigation hypothesis. J Affect Disord.

[B18] Keller MC, Nesse RM (2006). The evolutionary significance of depressive symptoms: different adverse situations lead to different depressive symptom patterns. J Pers Soc Psychol.

[B19] Nettle D (2004). Evolutionary origins of depression: a review and reformulation. J Affect Disord.

